# Family counseling after prenatal diagnosis of congenital heart disease: the role of a multidisciplinary and humanized approach

**DOI:** 10.61622/rbgo/2025EDT01

**Published:** 2025-07-15

**Authors:** Luciane Alves da Rocha Amorim, Edward Araujo Júnior

**Affiliations:** 1 Universidade Federal do Amazonas Manaus AM Brazil Universidade Federal do Amazonas, Manaus, AM, Brazil.; 2 Universidade Federal de São Paulo Escola Paulista de Medicina São Paulo SP Brazil Escola Paulista de Medicina, Universidade Federal de São Paulo, São Paulo, SP, Brazil.; 3 Faculdade Israelita de Ciências da Saúde Albert Einstein São Paulo SP Brazil Discipline of Woman Health, Faculdade Israelita de Ciências da Saúde Albert Einstein, São Paulo, SP, Brazil.; 4 Universidade Municipal de São Caetano do Sul São Caetano do Sul SP Brazil Discipline of Woman Health, Universidade Municipal de São Caetano do Sul, São Caetano do Sul, SP, Brazil.

The prenatal diagnosis of congenital heart disease (CHD) represents one of the most sensitive and impactful milestones in prenatal care. Beyond the technical aspect, it is a potentially disruptive event in the emotional, social, and spiritual life of the family. The way this information is delivered and the resources offered for support and guidance have a profound impact on the emotional processing of the news, the bonding between the parents and the baby, and decisions about pregnancy and neonatal care.

Although CHD is the most common fetal malformation and the leading cause of death due to congenital anomalies in the first year of life,^(1,2)^ scientific literature remains scarce regarding the structuring of family counseling in this context.^(3,4)^ In a scoping review conducted by our research group, we performed a comprehensive search in eight databases, including Medline, Embase, Scopus, Web of Science, LILACS, SciELO, PsycINFO, and Google Scholar. After screening 3,719 articles, only 21 met the inclusion criteria.^(3)^ This result alone highlights the lack of structured knowledge on the topic. Among the 21 included studies, most were from high-income countries, predominantly the United States, Sweden, and Canada. No studies were identified from Latin America, Africa, or other low- and middle-income regions, revealing a serious knowledge gap in contexts where access to healthcare is historically more limited. The methodologies varied, with a predominance of qualitative and cross-sectional descriptive studies. Only one randomized clinical trial was identified. The small number of studies and low methodological diversity hinder the development of standardized, scalable recommendations.^(3)^

Another significant point is the absence of validated and widely used instruments to assess the quality of family counseling following a prenatal diagnosis of CHD. In other words, although counseling is recognized as an essential step in perinatal care, there are no systematic tools that allow for comparison, measurement, or standardization of this practice across different centers or settings. This contrasts with other areas of fetal medicine, such as genetic counseling, where established protocols and checklists already exist.^(5,6)^ The absence of such tools limits the implementation of effective public policies and the evaluation of the quality of care.

In our study, we grouped the literature findings into four major themes: obstacles, strengths, opportunities, and challenges.^(3)^ The most frequent obstacles included difficulties in medical communication, limited consultation time, the use of excessively technical language, and the absence of systematic psychological support. Strengths were related to the presence of multidisciplinary teams, the use of explanatory visual aids, and the empathy shown by professionals when delivering the diagnosis. Opportunities included the potential for specialized training in counseling, the use of technology to expand access to care, and the creation of locally adapted protocols. The main challenges were the lack of structured services, inequality in access, absence of national guidelines, and weak community support networks. Analyzing these dimensions, it is clear that family counseling must go beyond simply providing information: it needs to be a continuous, empathetic process centered on the family and adapted to its social, cultural, and emotional context. It is essential that professionals involved be prepared not only with technical knowledge, but also with communication skills and ethical sensitivity to handle the emotional impact of such a sensitive diagnosis. Health professionals must be willing to listen, to explain in accessible language, to repeat information when necessary, and to respect each family's individual pace.

In our experience with families in the Northern region of Brazil, the challenges are further intensified.^(7)^ This is a region marked by vast geographic distances, high socioeconomic vulnerability, a shortage of specialists, and chronic deficits in advanced diagnostic equipment. Many diagnoses are made late or inaccurately, and referrals to reference centers are not always feasible. In this scenario, family counseling often represents not only a moment of support, but sometimes the only opportunity to prepare for a complex, demanding, and potentially high-risk birth. The quality of this counseling can determine treatment adherence, the family's coping capacity, and the emotional bonding between parents and their baby.

Furthermore, it is urgent to reflect on the role of public policies and national guidelines.^(1,2,8)^ The absence of clear protocols adapted to the Brazilian context hinders the standardization of counseling and compromises equity in healthcare. In countries like Brazil—with continental dimensions and significant regional inequalities—it is unacceptable for counseling to depend solely on the individual experience of each professional. We need national guidelines that promote best practices, ensure the presence of a multidisciplinary team whenever possible, and incorporate educational and evaluative tools to support healthcare professionals.

Professional training is another crucial point. Traditional medical education still devotes little attention to the communication of bad news, emotional support for patients and families, and the humanized approach to complex situations. Incorporating disciplines and training focused on these competencies into undergraduate and residency curricula could profoundly transform how counseling is conducted.^(9,10)^ Empathy, active listening, and the ability to deal with another's suffering are not innate traits, but clinical competencies that can—and must—be taught.

The integration of spirituality into the counseling process also proved to be a relevant factor in our studies. For many families, spirituality offers support, meaning, and resilience in the face of suffering.^(11)^ Acknowledging this dimension—while respecting diverse beliefs and offering qualified listening—can strengthen the bond between the healthcare team and the family, promoting more conscious and supported decisions. This does not imply imposing religious views but rather valuing what holds meaning for the family as part of holistic care.

Finally, the use of telehealth represents a strategic tool to expand access to specialized counseling, especially in remote areas.^(12–15)^ Well-structured videoconferencing platforms can enable families to receive expert guidance even in places without local coverage. This can reduce the time between diagnosis and intervention, avoid unnecessary travel, and extend the reach of best practices in counseling. Experiences with digital health technologies in other pediatric areas have already shown significant benefits in cost, time, and user satisfaction. Fetal cardiology must be integrated with these innovations.

In summary, prenatal diagnosis of congenital heart disease demands a response that goes beyond the technical: it requires mobilizing sensitivity, communication, psychological support, social networks, and integrated health policies. Family counseling is neither a luxury nor an optional step—it is an ethical and technical necessity, essential for promoting family-centered care. The future of fetal cardiology therefore demands not only investments in equipment and diagnostics, but also in people capable of transforming information into comfort, fear into preparedness, and solitude into shared strength.
